# Notopterol Attenuates Monocrotaline-Induced Pulmonary Arterial Hypertension in Rat

**DOI:** 10.3389/fcvm.2022.859422

**Published:** 2022-06-03

**Authors:** Lin Huang, Huayang Li, Suiqing Huang, Shunjun Wang, Quan Liu, Li Luo, Shuangjiao Gan, Guangguo Fu, PeiYun Zou, Guangxian Chen, Zhongkai Wu

**Affiliations:** ^1^Department of Cardiac Surgery, The First Affiliated Hospital of Sun Yat-sen University, Guangzhou, China; ^2^GuangZhou Janus Biotechnology Co. Ltd., Guangzhou, China

**Keywords:** pulmonary arterial hypertension, notopterol, pulmonary vascular remodeling, inflammation, pulmonary arterial smooth muscular cell

## Abstract

**Introduction:**

Current targeted pulmonary arterial hypertension (PAH) therapies have improved lung hemodynamics, cardiac function, and quality of life; however, none of these have reversed the ongoing remodeling of blood vessels. Considering notopterol, a linear furocoumarin extracted from the root of traditional Chinese medicine Qiang-Huo (*Notopterygium incisum*), had shown the antiproliferative and anti-inflammatory properties in previous studies, we hypothesized that it could play a role in ameliorating PAH.

**Methods:**

*In vivo*, we conducted monocrotaline (MCT) induced PAH rats and treated them with notopterol for 3 weeks. Then, the rats were examined by echocardiography and RV catheterization. The heart and lung specimens were harvested for the detection of gross examination, histological examination and expression of inflammatory molecules. *In vitro*, human pulmonary arterial smooth muscle cells (HPASMCs) were treated with notopterol after hypoxia; then, cell proliferation was assessed by cell counting kit-8 and Edu assay, and cell migration was detected by wound healing assays.

**Results:**

We found that notopterol improved mortality rate and RV function while reducing right ventricular systolic pressure in MCT-induced PAH rats. Furthermore, notopterol reduced right ventricular hypertrophy and fibrosis, and it also eased pulmonary vascular remodeling and MCT-induced muscularization. In addition, notopterol attenuated the pro-inflammatory factor (IL-1β, IL-6) and PCNA in the lungs of PAH rats. For the cultured HPASMCs subjected to hypoxia, we found that notopterol can inhibit the proliferation and migration of HPASMCs.

**Conclusion:**

Our studies show that notopterol exerts anti-inflammatory and anti-proliferative effects in the pulmonary arteries, which may contribute to prevention of PAH.

## Introduction

Pulmonary arterial hypertension (PAH) is a chronic and severe cardiopulmonary syndrome caused by cell proliferation and fibrosis of the small pulmonary arteries, leading to a progressive increase in pulmonary vascular resistance (1). The prevalence of PAH in all its forms is about 15–50 per million people and the annual incidence is 2.4 per million people (2). Current targeted therapies, alone or in combination, could improve pulmonary vascular function and hemodynamics, and reduce hospitalization rates (3). However, with the exception of epoprostenol, which improved survival of WHO functional grade IV PAH patients, other agents did not reduce mortality (4). None of these treatments have addressed the ongoing loss and remodeling of blood vessels and long-term outcomes remain poor (5).

Pulmonary vascular remodeling is a characteristic of the pathophysiological process of PAH, including the abnormal proliferation of pulmonary artery smooth muscle cells (PASMCs) and fibroblasts as well as the endothelial mesenchymal transformation of pulmonary artery endothelial cells (6). A large number of previous studies have found that many traditional herbal ingredients, such as thymoquinone, formononetin, and resveratrol, could effectively inhibit the proliferation of smooth muscle cells to significantly attenuate pulmonary vascular remodeling in animal models (7–9). In addition, the vascular remodeling process of PAH is closely related to both innate and adaptive immune system (10). Excessive infiltration of perivascular inflammatory cells and the release of local inflammatory factors lead to excessive proliferation and phenotypic transformation of different vascular cells, causing changes in the three-layer structure of vascular wall (11). Notopterol, a kind of linear furancoumarin, is an active compound isolated from the rhizome of *Notopterygium incisum* Ting ex H. T. Chang (12). Previous studies have reported that notopterol showed preliminary antiproliferative effects on some cancer cell line, such as HEPG-2, McF-7, and C6, etc. (12). Additionally, it can also inhibit proliferation, apoptosis, differentiation, and cell cycle of human acute leukemia HL-60 cells (13). Apart from that, notopterol treatment reduced synovitis and structural cartilage and bone damage, reduced the number of F4/80^+^ and iNOS^+^ inflammatory macrophages in synovial tissue of mice with collagen-induced arthritis, and inhibited the production of IL-1β, TNF-α, and IL-6 proinflammatory factors in the lesion and circulation (14). Considering the antiproliferative and anti-inflammatory properties of notopterol on experimental animal and cell lines, we therefore hypothesized that notopterol could play a similar role in ameliorating the progression of PAH of monocrotaline-injected rats.

To test this hypothesis, *in vivo*, we examined monocrotaline-induced PAH rats and treated them with notopterol. Particularly, we discussed whether notopterol played a beneficial role in inhibiting proliferation and inflammation of PASMCs. *In vitro*, we investigated the effect of notopterol on the proliferation and migration of PASMC under hypoxia environment.

## Method

### Animal Experiments

To exclude the influence of gender on the results, male Sprague-Dawley rats with body weight of about 220–250 g were selected (animals were from experimental Animal Center, Sun Yat-Sen University, Guangzhou, China, SCXK2018-0008). Notably, eight rats were given a single subcutaneous injection of saline (normal: *n* = 8) on day 1. In addition, sixteen rats were given a single subcutaneous injection of MCT (60 mg/kg; Sigma, St Louis, MO, United States) on day 1, and its effect lasted for 3 weeks without supplementation. Rats injected with MCT were randomly divided into two groups (MCT + vehicle: *n* = 8; MCT + notopterol: *n* = 8). From 1 to 21 days, rats in the MCT + notopterol group were intragastric with nototerol (20 mg/kg/day, Keyuan Biology, Guangzhou, China) daily, and rats in the MCT + vehicle group were intragastric with vehicle [1% DMSO + 10% Tween 80+ 0.5% sodium cellulose +89% saline] daily.

### Echocardiogram

The rats were anesthetized by intraperitoneal injection of pentobarbital sodium (50 mg/kg), and the heart rate of the rats was maintained at approximately 300 beats/min. Transthoracic echocardiography was performed with a 25 MHz linear array transducer (Vevo 2100, Visual Sonics, Toronto, Canada). Pulmonary acceleration time (PAT) and peak ejection time (PET) were measured by right ventricular outflow tract pulse Doppler recording. Left ventricular ejection fraction (LVEF) was measured by short-axis M-mode ultrasound recording. The analysis was performed among observers blinded to the source of the images.

### RVSP Measurement and Hypertrophy Index

After echocardiography, the rats were anesthetized (pentobarbital sodium, 50 mg/kg), and the right ventricular pressure (RVP) was measured by right ventricular catheter with terminal invasive hemodynamic examination. The rats were fixed on a plank. The right jugular vein was then separated and intubated. A PE-50 tube filled with heparin saline was connected to a pressure sensor (Techman, Chengdu, China) and inserted into the right external jugular vein. The smooth appearance of the ventricular pressure waveform indicates that the catheter has reached the right ventricle. RVP was recorded and systolic pressure (RVSP) was analyzed. After RVSP measurement, chest was quickly opened, and the left and right atria as well as blood vessels along the atrioventricular junction were cut off. The heart and lung specimens were taken after rinsing with saline solution. Right ventricle, left ventricle, and interventricular septum were separated and weighed separately. The RV hypertrophy index was calculated as [RV/(LV + IVS)].

### Masson Staining and Hematoxylin-Eosin (H&E) Staining

After hemodynamic measurements and sampling, heart and lung specimens were harvested for morphometry and histological analysis. The heart and the middle lobe of the right lung were dissected, fixed with 4% paraformaldehyde for 24 h, embedded in paraffin, and sectionalized. The heart sections were Masson trichromatic staining and the lung sections were H&E staining. Light microscopy (Carl Zeiss, Jena, Germany) is used for overall histological evaluation. RV fibrosis was evaluated by the ImageJ software (image-J.1.5V). The calculation formula of pulmonary artery wall thickness (WT) is as follows: WD/TD (%) = vessel wall thickness/vessel external diameter × 100.

### Immunohistochemical Staining

Immunohistochemical staining was used to detect the middle lobe of the right lung expression of smooth muscle actin (α-SMA). Tissue slides were degreased with xylene and then continuously rehydrated with ethanol. After brief proteolysis and peroxidase blocking, the slides were mixed with antibodies of α-SMA (19,245s, Cell Signaling Technology, United States, 1:500) and were then incubated overnight at 4°C. The slides were washed first to remove the unbound primary antibody, and then incubated with the peroxidase bound secondary antibody. The specific binding secondary antibody was detected by Dako Envision detection system (Dako, Glostrup, Denmark).

### Immunoblotting

Rapid freezing of the lower lobe of the right lung was homogenized and lysed with RIPA lysis buffer (Beyotime Biotechnology). The protein content was determined by BCA protein assay (Thermo Fisher Scientific, Waltham, MA, United States). The same amount of protein was dissolved in SDS-polyacrylamide gel electrophoresis (PAGE) (10%) and transferred to polyvinylidene fluoride (two) membrane. After blocking in 5% bovine serum albumin (BSA) and Tris-buffered saline for 1 h, the membrane was incubated with the required primary antibody at 4°C for 12 h. The membrane was then treated with an appropriate horseradish peroxidase bound secondary antibody (Cell Signaling Technology, Danvers, MA, United States), and the immune response bands were detected with chemiluminescence (ECL) reagent (Merck Millipore, Billerica, MA, United States). Specific antibodies against PCNA (13,110, Cell Signaling Technology, Danvers, MA, United States) IL-1 (Affinity Biosciences Cat# AF5103), IL-6 (Affinity Biosciences Cat# DF6087), AKT (4,685, Cell Signaling Technology, Danvers, MA, United States), phosphorylated AKT (4,060, Cell Signaling Technology, Danvers, MA, United States), mTOR (2,983, Cell Signaling Technology, Danvers, MA, United States), phosphorylated mTOR (5,536, Cell Signaling Technology, Danvers, MA, United States), IKB α (4,814s, Cell Signaling Technology, Danvers, MA, United States), phosphorylated IKK α/β (2,697s, Cell Signaling Technology, Danvers, MA, United States), p65 (sc-8008, Santa Cruz, CA, United States), STAT3 (9,139, Cell Signaling Technology, Danvers, MA, United States), phosphorylated STAT3 (9,145, Cell Signaling Technology, Danvers, MA, United States), and GAPDH (60004-1-lg, Protetech, Chicago, IL, United States). Objective protein expression was normalized to GAPDH for analysis.

### Quantitative Real-Time PCR (qRT-PCR)

Total RNA was extracted from tissue homogenates using Universal RNA purification kit (EZBioscience, Roseville, MN, United States) and converted to cDNA with 2 × Color SYBR Green qPCR Master Mix (ROX2 plus) (EZBioscience, Roseville, MN, United States). RNA concentration was measured using NanoDrop2000 spectrophotometer (Thermo Fisher Scientific, Waltham, MA, United States). Reverse transcription uses Color Reverse Transcription Kit (with gDNA Remover) (EZBioscience, Roseville, MN, United States) for each reaction. Then, qRT-PCR was performed on the Light Cycler 480 system. The list of primers with their sequences is as follows: for Collagen I, forward primer: GTACATCAGCCCAAACCCCA, reverse primer; TCGCTTCCATACTCGAACTGG; for GAPDH, forward primer; CGCTAACATCAAATGGGGTG, reverse primer; CGCTAACATCAAATGGGGTG. For analysis, the expression of target genes was normalized to GAPDH.

### Cell Culture

Human pulmonary artery smooth cells (HPASMCs, ScienCell, Carlsbad, CA, United States) were cultured at 37°C and 5% CO_2_ with Dulbecco's modified Eagle's medium (DMEM) containing 10% fetal bovine serum (FBS) and 1% penicillin/solution (Gibco, Grand Island, NY, United States). For *in vitro* experiments, HPASMCs between passages three and eight were used.

### EdU Proliferation Assay

Cell proliferation was assessed with an EdU Cell Proliferation assay kit (EDU-555, Beyotime, Shanghai, China) according to the manufacturer's instruction. HPASMCs were seeded onto 2 × 10^4^ cells/well in 24-well plates and cultured overnight. Then, the culture medium was changed, and 5, 10, or 20 μM of notopterol was added to the culture medium. The cells were incubated under hypoxia (2% oxygen) or normoxia (21% oxygen) at 37°C for 36 h, and then 10 μM of EdU was added. After 2 h incubation, the culture medium was discarded and the cells were fixed in 4% paraformaldehyde at room temperature for 15 min. After aspiration of the fixing solution, the cells were stained using the BeyoClick™EdU Cell Proliferation Kit with Alexa Fluor 555 and DAPI (nuclear staining). The results were then observed through a fluorescence microscope (Leica, Wetzlar, Germany). The ImageJ software (National Institutes of Health, United States) was used to quantify the results.

### Cell Viability Analysis

Cell viability was detected using Cell Counting Kit-8 (CCK-8, Beyotime, Shanghai, China). In brief, HPASMCs concentration of 6 × 10^3^ cells/well was inoculated in 96-well plates and cultured overnight. HPASMCs were exposed to hypoxic (2% oxygen) or normoxic (21% oxygen) conditions at 37°C for 36 h with different concentrations of notopterol. Then, the culture medium was discarded, 10 μl CCK-8 solution was added to each well, and the plates were incubated for 2 h at 37°C. Optical density values at 450 nm were measured using a microplate meter (Thermo Fisher Scientific, Waltham, MA, United States).

### Wound Healing Assay

Wound healing assay was used to assess migration of PASMCs. About 4 × 10^5^ cells were inoculated in six-well plates and cultured in complete DMEM medium until fusion. The tip of the 200 μl pipette was directly sliced through the monolayer of cells to obtain a wide noncellular area and washed with PBS three times to remove cell debris caused by the scratches. The scratch areas of each group were observed and recorded under inverted phase-contrast microscope at 0 and 24 h, and the scratch areas were measured by the ImageJ software (National Institutes of Health, United States).

### Statistical Analysis

All data were presented as mean ± EM. All statistical analyses were performed using the GraphPad Prism software (Version 8, La Jolla, CA, United States). Univariate ANOVA was used for comparison between the groups, followed by Tukey *post-hoc* test. If *p* < 0.05, the difference was considered statistically significant.

## Results

### Notopterol Improves the Mortality Rate and RV Function While Reducing RVSP in PAH Rats

**Figure 1:**
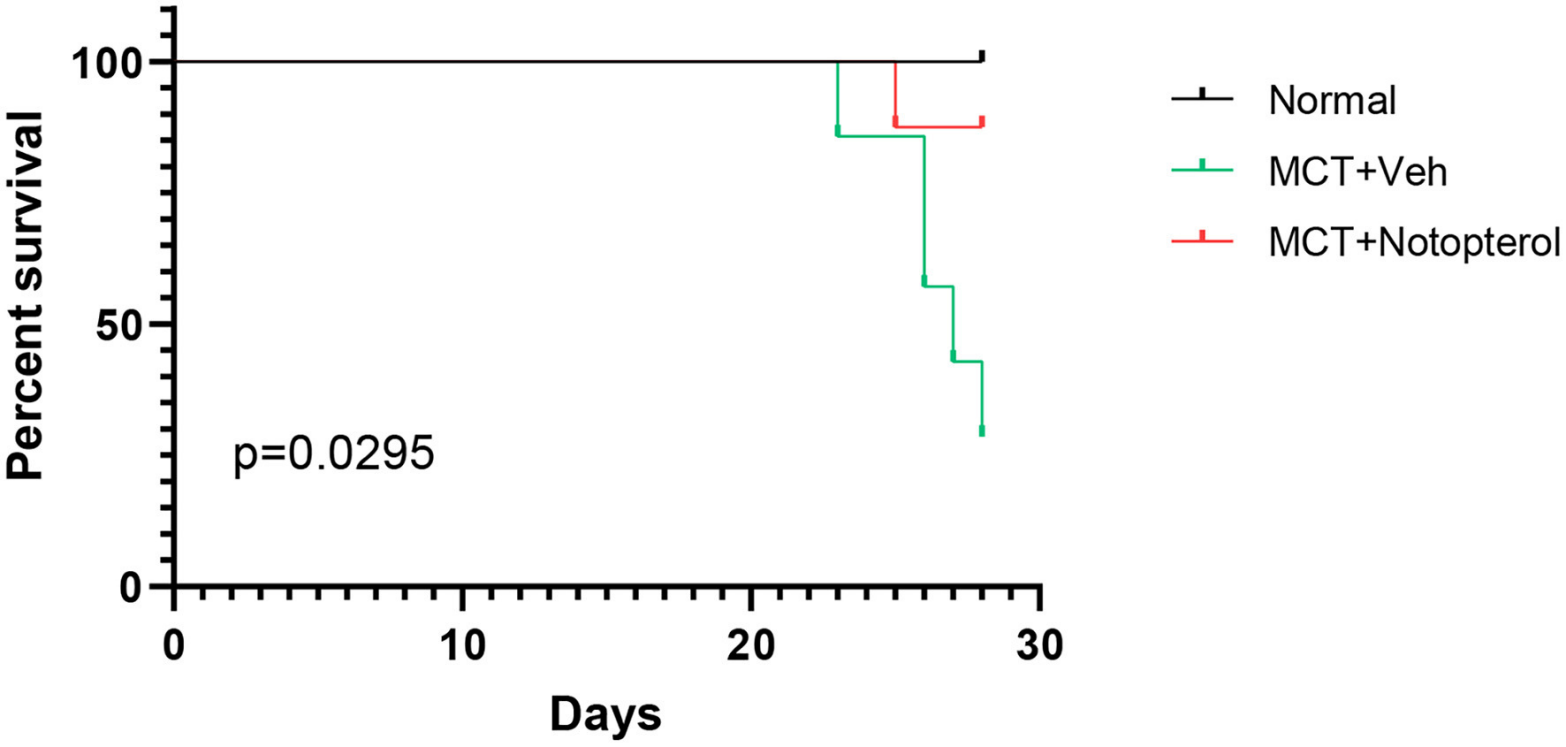
Notopterol improves mortality rate in pulmonary arterial hypertension rats. Kaplan–Meier survival curves in the group (*n* = 10 for each group).

**Figure 2:**
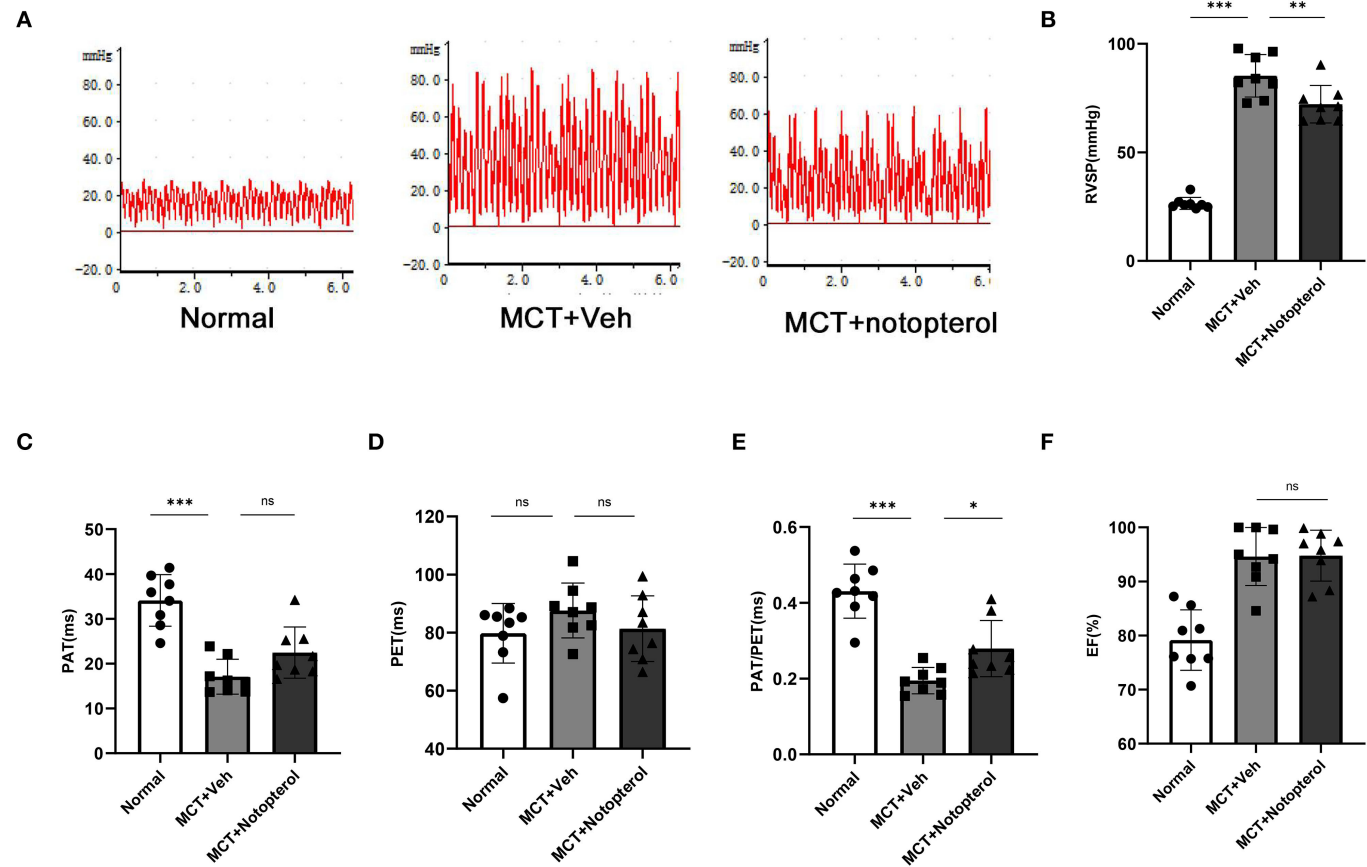
Notopterol improves right ventricular function and reduces right ventricular systolic pressure in pulmonary arterial hypertension rats. **(A)** The right ventricular pressure waveform. Quantification of RVSP, **(B)** PAT, **(C)** PET, **(D)** PAT/PET, **(E)** LVEF, **(F)** 3 weeks after MCT injection (*n* = 8 for each group); *p < 0.05, **p < 0.01, ***p < 0.001. MCT, monocrotaline; Veh, vehicle; PAT, pulmonary blood flow acceleration time; PET, pulmonary ejection time; PAT/PET, pulmonary blood flow acceleration time/ pulmonary ejection time; RVSP, right ventricular systolic pressure.

Within 4 weeks after MCT injection, ten rats died, seven of which were from the vehicle group, and the mortality of the notopterol group was significantly lower than that of the vehicle group (Figure 1). Due to the high mortality rate of the 4 weeks model, we set the end point of the experiment to be 3 weeks after MCT injection. RVSP raised significantly in the vehicle group compared with the normal group (85.28 ± 9.763mmHg vs. 26.52 ± 2.73 mmHg, *p* < 0.05; Figures 2A,B), indicating that the PAH was successfully established. It could be seen that treatment with notopterol significantly decreased RVSP compared with the vehicle group (72.17 ± 8.66 mmHg vs. 85.28 ± 9.76 mmHg, *p* < 0.05). Cardiac ultrasound showed decreased pulmonary blood flow acceleration time (PAT) and pulmonary blood flow acceleration time/pulmonary ejection time (PAT/PET) in vehicle groups compared with the normal group (PAT: 22.47 ± 5.72 ms vs. 34.08 ± 5.76ms, PAT/PET: 0.27 ± 0.07 vs. 0.43 ± 0.07, *p* < 0.05; Figures 2C–E). PAT/PET increased significantly in the notopterol group compared with the vehicle group (0.28 ± 0.07 ms vs. 0.19 ± 0.03 ms, *p* < 0.05), although there was no significant difference in left ventricular ejection fraction (LVEF) between the vehicle group and the notopterol group (Figure 2F).

### Notopterol Reduces Right Ventricular Hypertrophy and Fibrosis in PAH Rats

**Figure 3:**
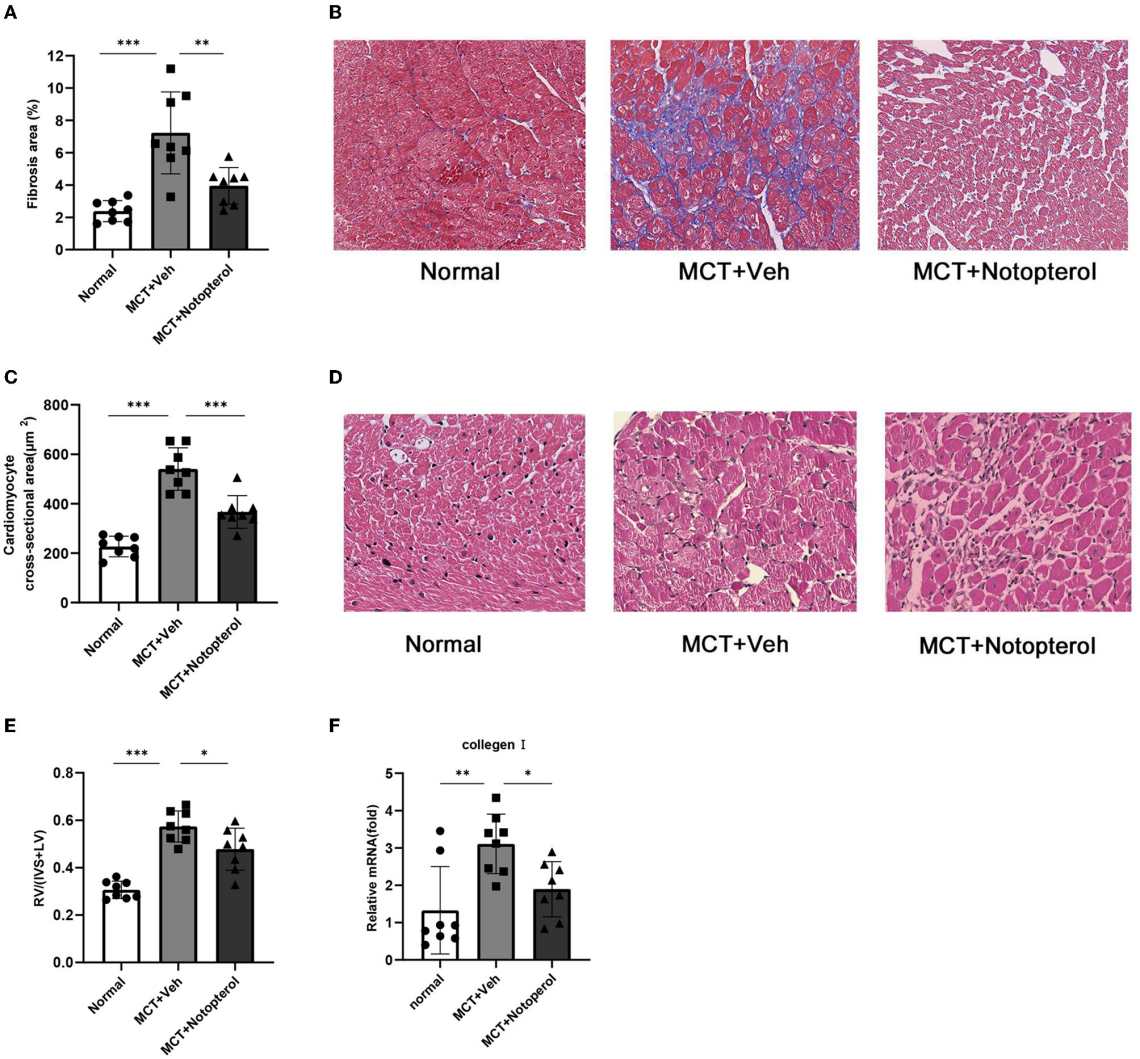
Notopterol reduces right ventricular hypertrophy and fibrosis in pulmonary arterial hypertension rats. **(A)** Quantification of Masson staining; **(B)** Representative histopathological images of RV sections with Masson staining for collagen deposition (40 ×); **(C)** Quantification of Myocyte area; **(D)** Myocyte area. Representative cross-section cardiomyocytes (40 ×); **(E)** RV hypertrophy index; **(F)** RV mRNA expression of collagen I. (*n* = 8 for each group); *p < 0.05, **p < 0.01, ***p < 0.001. IVS, interventricular septum; LV, left ventricle.

In PAH rats, remodeling and hypertrophy increased due to an increase in RV pressure, which showed a higher right ventricular hypertrophy index (RVHI). The RVHI of the notopterol group was significantly lower than that of the vehicle group (0.47 ± 0.08 vs. 0.57 ± 0.06, *p* < 0.05; Figure 3E). Masson staining of the RV specimens showed that the fibrosis fraction of RV (Figure 3B) in the vehicle group was significantly increased compared with the normal rats (7.22 ± 2.53 vs. 2.38 ± 0.64%, *p* < 0.05), although notopterol treatment reduced the fibrosis fraction of the RV significantly (3.944 ± 1.13 vs. 7.226 ± 2.534%, *p* < 0.05; Figure 3A). HE staining of the RV specimens showed that the cross-sectional area of cardiomyocytes of RV (Figure 3D) in the vehicle group was increased compared with normal rats (226.8 ± 41.50 μm^2^ vs. 540.2 ± 86.04 μm^2^; *p* < 0.001), though notopterol treatment reduced the myocytes area of the RV (540.2 ± 86.04 μm^2^ vs. 366.5 ± 66.25 μm^2^; *p* < 0.001; Figure 3C) significantly. Furthermore, mRNA expression of type I collagen in the RV was increased in pulmonary hypertension rats, but notopterol treatment significantly reduced its expression (Figure 3F).

### Oral Administration of Notopterol Eases Pulmonary Vascular Remodeling

**Figure 4:**
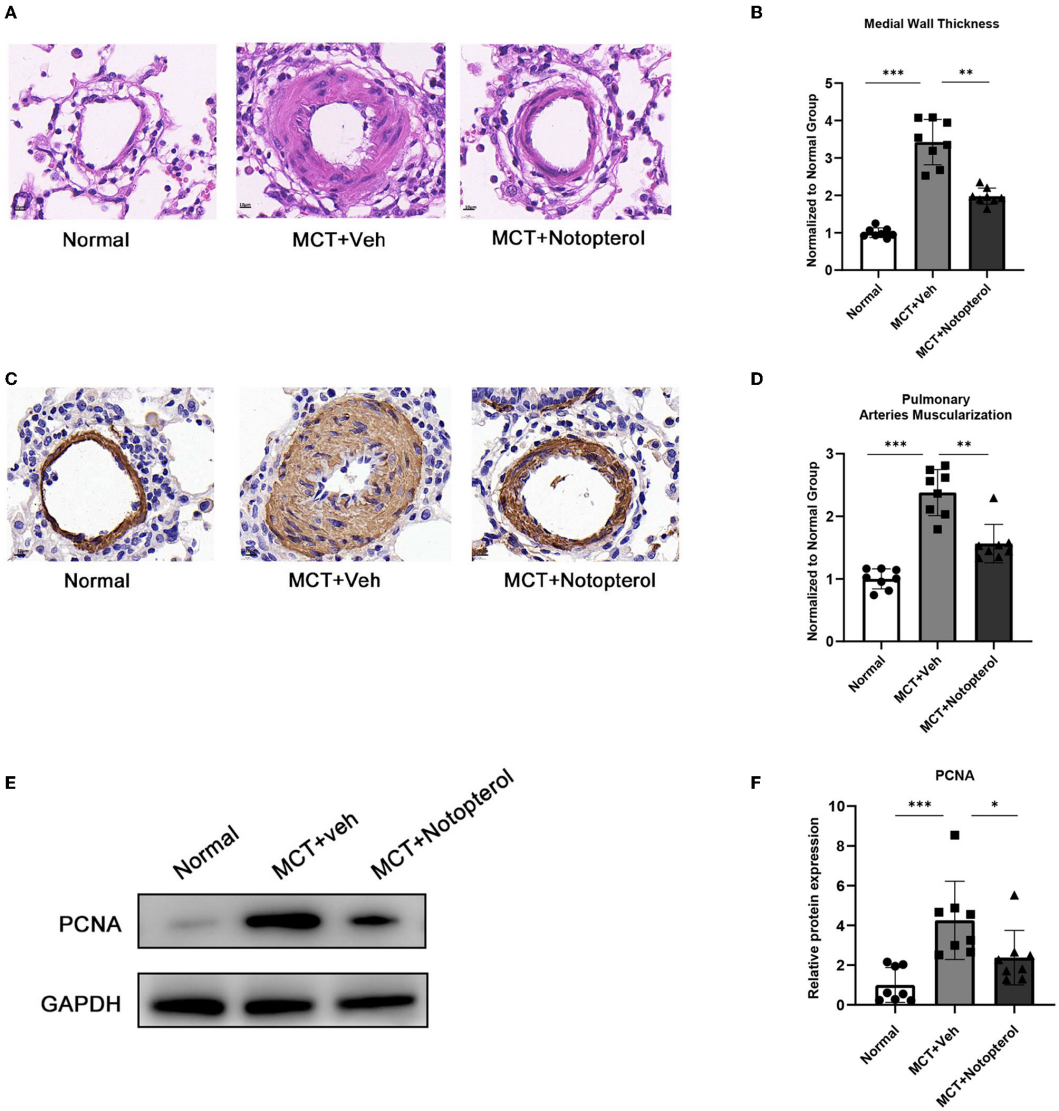
Notopterol eases pulmonary vascular remodeling. **(A)** Representative H&E staining micrograph of lung tissue sections (100 ×); **(B)** Quantification of the medial wall thickness of the small pulmonary arteries (*n* = 8 for each group); **(C)** Expression of α-SMA in lungs evaluated with immunohistochemistry staining (100 ×); **(D)** Quantification of the arteries muscularization of the small pulmonary arteries (*n* = 8 for each group); **(E)** Western blots of PCNA in lungs; **(F)** Quantification of PCNA expression in the lungs. (*n* = 4 for each group). *p < 0.05, **p < 0.01, ***p < 0.001. H&E, hematoxylin-eosin; α-SMA, α-smooth muscle actin; PCNA, proliferating cell nuclear antigen.

One of the mechanisms by which MCT causes PAH in rats is that it can contribute to pulmonary vascular remodeling, manifested by an increase in the thickness of the small pulmonary artery wall (15). HE staining of lung specimens demonstrated that notopterol reduced the increase of pulmonary small vessel thickness induced by MCT (1.97 ± 0.21 vs. 3.42 ± 0.60, Figures 4A,B). Immunohistochemistry with α-SMA showed that the degree of muscular activity of pulmonary arterioles was increased in PAH rats, which was attenuated by notopterol treatment (2.38 ± 0.36 vs. 1.56 ± 0.31, Figures 4C,D). As over-proliferation of PASMC plays an important role in pulmonary vascular remodeling, we detected the expression of proliferating cell nuclear antigen (PCNA) in the lung specimens. We found that PCNA increased significantly in lung tissue of MCT-induced PAH, and decreased after notopterol treatment (*p* < 0.05, Figures 4E,F).

### Notopterol Attenuates the Pro-inflammatory Factors in the Lungs of PAH Rats

**Figure 5:**
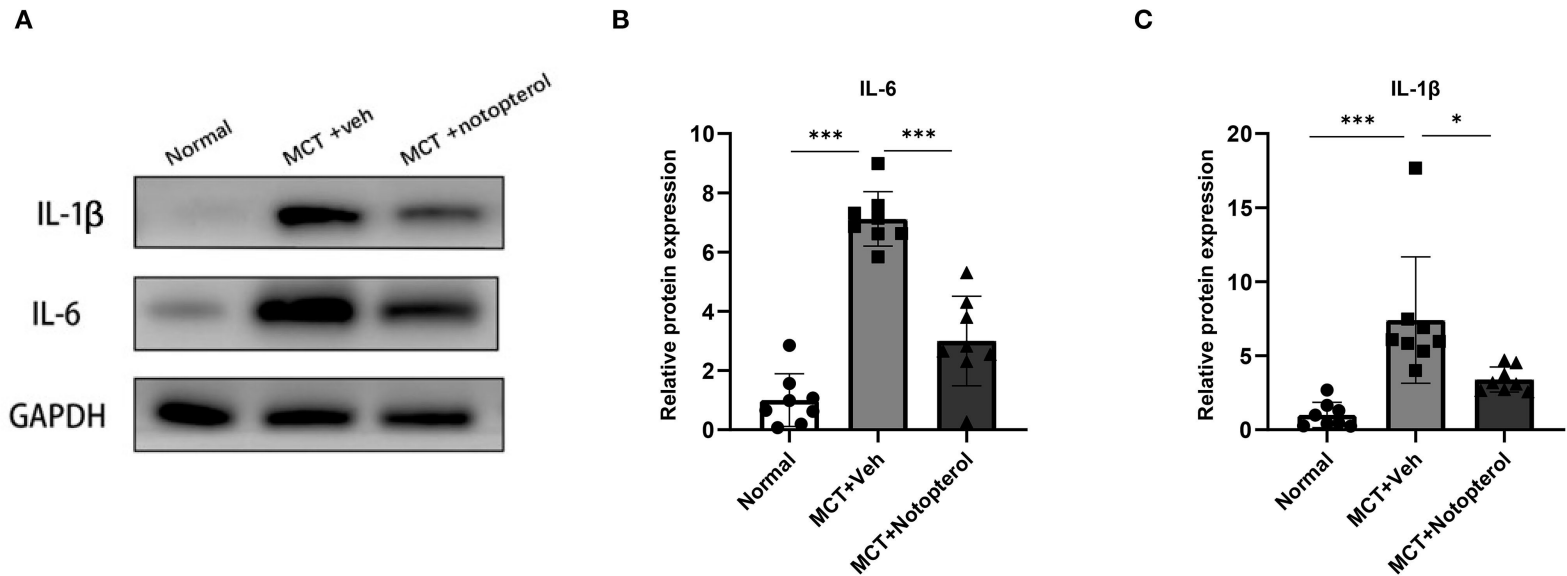
Notopterol attenuates the pro-inflammatory factors in the lungs of PAH rats. **(A)** Western blots of IL-1β and IL-6 in lungs; **(B)** Quantification of IL-1β expression in the lungs (*n* = 8 for each group); **(C)** Quantification of IL-6 expression in the lungs (*n* = 8 for each group). *p < 0.05, ***p < 0.001.

To examine whether notopterol treatment affected MCT-induced pulmonary inflammatory infiltration, we extracted protein from the lung tissue specimens for Western blot detection. As shown in Figures 5A–C, the protein levels of IL-1β and IL-6 in the lungs of model rats were significantly higher than that of the normal group, but this increase can be reversed by notopterol treatment (all *p* < 0.05).

### Notopterol Inhibits the Proliferation and Migration of HPASMCs in Response to Hypoxia *in vitro*

**Figure 6:**
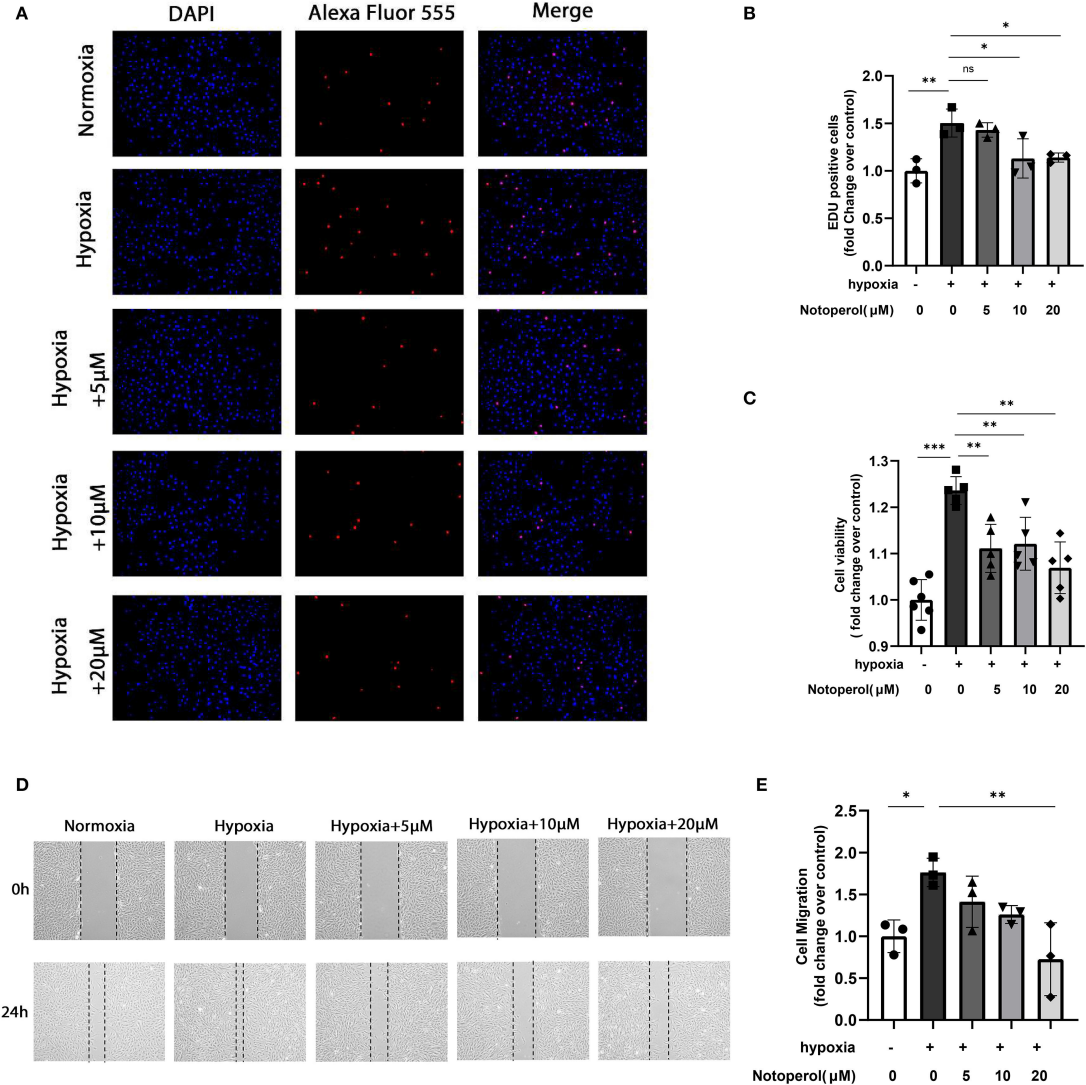
Notopterol inhibits the proliferation and migration of human pulmonary artery smooth muscle cells in response to hypoxia *in vitro*. **(A)** Representative images of the EdU assay of HPASMCs *in vitro* (100 ×); **(B)** Quantification of the EdU assay of HPASMCs; **(C)** CCK-8 assay of HPASMCs; **(D)** Representative images of the wound healing assay of HPASMCs *in vitro*; **(E)** Quantification of the wound healing assay of HPASMCs; (*n* = 3, biological replicates; three wells each time for the EdU assay, six wells each time for the CCK-8 assay, three wells each time for the wound healing assay). *p < 0.05, **p < 0.01, ***p < 0.001. EdU, 5-ethynyl-2′-deoxyuridine; HPASMCs, human pulmonary arterial smooth muscle cells; CCK-8, Cell Counting Kit-8.

Proliferation and migration of PASMCs promote pulmonary vascular remodeling, which leads to irreversible progression of PAH (16, 17). Therefore, we investigated the effects of notopterol on the proliferation and migration potential of HPASMCs cultured *in vitro*. EDU assay showed that hypoxia could significantly promote the proliferation of HPASMCs, and notopterol treatment showed anti-proliferative effect at the concentrations of 10 and 20 μM (all *p* < 0.05, Figures 6A,B). Similarly, CCK-8 analysis showed that it inhibited the cellular viability under hypoxia after the notopterol (5, 10, and 20 μM) treatment without dose-dependent effect (all *P* < 0.05, Figure 6C). Wound healing experiments showed that the migration of HPASMCs could be promoted in hypoxic environment, and notopterol inhibited the migration of HPASMCs significantly (Figures 6D,E).

## Discussion

This study reported that notopterol had new and previously unrecognized protective effects on experimental PAH. In the MCT-treated rats, the increase of RVSP after 3 weeks of MCT administration was in parallel with pulmonary artery remodeling, right ventricular dysfunction, and myocardial fibrosis. Notopterol improved the mortality rate, alleviated MCT-induced PAH, and reduced pulmonary vascular remodeling along with MCT-induced muscularization as well as vascular cell proliferation. It improved the RV dysfunction, hypertrophy, and myocardial fibrosis in rats. In addition, it can also reduce the protein levels of proinflammatory factor IL-1β and IL-6 in the lung tissue. Beyond that, for the cultured HPASMCs subjected to hypoxia, we found that notopterol could inhibit the proliferation and migration of PASMCs.

In this study, we used MCT injection to induce PAH, which has similar characteristics to human diseases in hemodynamic changes, endothelial dysfunction, vascular remodeling, and RV failure, without direct liver or heart injury (18). We obtained a model by subcutaneous injection of MCT for 4 weeks, and administered notopterol at the same time. We found that ten rats died before the end of the study, of which seven were in the vehicle group, and there were pleural effusion and ascites after anatomy, possibly owing to the RV failure possibly caused by severe PAH. As the rats in the 4 weeks model vehicle group had obvious pulmonary hypertension, extremely poor RV function, and difficulties in follow-up manometry, ultrasound, and material acquisition, we injected rats again with MCT for 3 weeks, which were used in the latter experiments.

Notopterol showed anti-proliferative and anti-inflammatory effects in previous studies. In human acute leukemia HL-60 cells (13). Notopterol could inhibit proliferation and cell cycle, and promote apoptosis and cell differentiation. Notopterol also has anti-proliferative activity against human hepatoma (HepG-2) and C6 cell lines (12). The abnormal proliferation of PASMCs plays an important role in pulmonary vascular remodeling, a characteristic of the pathophysiological process of PAH (6). In addition, in the mice with collagen-induced arthritis, notopterol treatment reduced synovial inflammation along with structural cartilage and bone damage, decreased the number of F4/80^+^ and iNOS^+^ inflammatory macrophages, and inhibited the production of IL-1β, TNF-α, and IL-6 in the lesion and circulation (14). Also, many studies have reported that the vascular remodeling process of PAH was closely related to perivascular inflammation (19). However, the effect of notopterol on PAH has not been studied in the past (12–14, 20, 21). At present, it is considered that although the pathogenic factors of PAH may be idiopathic, genetic, or other factors, pulmonary vascular remodeling is the main pathological feature of PAH (22). The unbalanced proliferation, migration, and apoptosis of PASMCs can lead to the continuous increase of pulmonary vascular resistance (22). Therefore, reversing vascular remodeling and inhibiting PASMC proliferation and migration have become new therapeutic concepts for PAH. Therefore, we inferred that notopterol could ameliorate the progression of PAH of MCT-injected rats by anti-proliferation and anti-inflammation. And our research revealed that the therapeutic effect of notopterol on PAH may be through these two ways. Additionally, compared with synthetic chemicals, natural compounds seem to have advantages in safety (18), and notopterol can be easily extracted from *N. incisum* Ting ex HT Chang with low long-term treatment cost (14). In conclusion, the drug may contribute to the treatment of PAH.

Inflammation plays a key role in the experimental model of PH and human PAH (23). In the experimental model of pulmonary hypertension, perivascular inflammation precedes pulmonary vascular remodeling (24). Previous studies have shown that inflammatory cells such as macrophages, dendritic cells, lymphocytes, and mast cells are involved in the progression of PAH. Proinflammatory factors such as IL-6 and IL-1β (25), and chemokines and their receptors, including CX3CL1 (26), CCR2, and CCR5 (27), play a key role in human PAH and PH in animal models. IL-6 and IL-1β can mediate pulmonary vascular remodeling and progressive pulmonary vascular occlusion (28). Literatures have shown that IL-6 and IL-1β could regulate the function of smooth muscle cells and endothelial cells, activate a series of inflammatory factors, and promote the abnormal proliferation and migration of PASMC (24, 28–32). Consistent with the previous literatures, our results showed that MCT injection can cause inflammation of pulmonary vessels, and the protein expression of IL-1β and IL-6 was upregulated, while notopterol treatment significantly reduced the protein levels of these pro-inflammatory factors. At the same time, MCT-induced pulmonary artery wall thickening and pulmonary artery muscularization were significantly alleviated in rats after notopterol treatment. In addition, the increase of proliferation marker PCNA induced by MCT also decreased significantly after notopterol treatment. We inferred that the remission of pulmonary inflammation is beneficial to the possible weakening of vascular cell proliferation, so as to lead to the reduction of pulmonary vascular remodeling, the decrease of RVSP, and the improvement of RV dysfunction. More importantly, in this severe PAH model, these benefits are associated with prolonged survival. Although the mechanism behind these benefits is still unknown, the effects of notopterol on the systemic changes in hemodynamics, or potential direct effects on myocardium, may help to reduce the severity of the disease.

Previous studies have pointed out that the proliferation and migration of PASMCs lead to the thickening of the middle membrane, the narrowing, or even occlusion of the vascular lumen, which plays a vital role in the occurrence and development of PAH (16, 17). Previous reports also pointed out that anti-proliferation is an important therapeutic target of PAH (28, 33). We have detected that notopterol can reduce PCNA expression in the lung specimens of MCT-treated rats. We wondered whether notopterol could play a role in proliferation of PASMCs. Our *in vitro* experiments proved that the proliferation and migration of HPASMCs under hypoxia can be inhibited by notopterol. This discovery showed that in addition to reducing the inflammatory processes in the lung induced by MCT, notopterol also reduces pulmonary artery remodeling by inhibiting PASMCs proliferation and migration, so as to alleviate pulmonary hypertension. Overall, our data suggested that notopterol reduced PA remodeling through anti-inflammatory and anti-proliferative effects, which helps to reduce downstream RV fibrosis and dysfunction, so as to improve hemodynamic measurement and reduce mortality in MCT-treated rats.

So far, we have found the role in MCT-induced PAH and HPASMCs under hypoxia that notopterol plays, but we still do not know the exact mechanism. Therefore, we explored which signaling pathway would be involved. In the pathways associated with PAH, we noted that Akt/mTOR, NF-κB, and JAK/STAT3 are common and important for anti-proliferation or anti-inflammation. PI3K/Akt/mTOR signaling pathway is a multifunctional pathway involved in the regulation of vascular remodeling, vasoconstriction, and macrophage polarization (34). The pro-proliferation and pro-survival effects of Akt/mTOR in PASMC have been confirmed in human PAH and in animal PH models (35). Beyond that, NF-κB belonging to the Reel protein family are transcription factors involved in cell proliferation, apoptosis, differentiation, and survival in PASMCs (36, 37). It also affects the expression of inflammatory factors such as IL-1 β, iNOS, MCP-1, ICAM, IL-6, IL-8 etc. (38, 39). In addition, the JAK/STAT activation is involved in the proliferation, migration, senescence, and transformation of endothelial cells and PASMCs into mesenchymal/myofibroblast cells (40). JAK2/STAT3 pathways are involved in the regulation of hypoxia-mediated vascular remodeling by inhibiting PASMC proliferation and hyperplasia (33, 40–42). Taking the role of the three pathways in inflammation or pulmonary vascular remodeling in PAH into account, we considered that notopterol may act through one of them. However, our results demonstrated no significant differences in the expression of phosphorylation of important proteins in the Akt/mTOR, NF-κB, and JAK2/STAT3 signaling pathways, which were observed before and after notopterol treatment on cellular models (refer to Supplementary Figure 1).

Although we have revealed the therapeutic effects of notopterol on PAH in inhibiting PASMCs proliferation and migration as well as attenuating perivascular inflammation, there are still some limitations in our research. First, this is a prevention study. Although there are many studies focusing on preventing the progress of animal models with PAH (43–45). We have testified that notopterol was effective for attenuating the advance of PAH. The regression effect of notopterol on PAH rats still needs to be supplied in the future research. Second, the molecular mechanisms of the protective effects of notopterol on PAH are not yet known. In this study, we detected a limited number of signaling pathway proteins by Western blot. However, the results were negative. In addition to proteins, we considered that non-coding RNA may play a role. Therefore, we also intended to use RNAseq to reveal the molecular mechanisms of the effects of notopterol on PAH in the future research. Third, although the previous study has reported that there was no detectable toxicity arising from notopterol treatment in mice (14). We do not know the reactions in rats. Therefore, the toxicity of notopterol in rats would be needed in the future study.

## Conclusion

Notopterol can inhibit PASMCs proliferation and migration, and reduce expression of IL-1β and IL-6 to reduce vascular remodeling of MCT-induced PAH rat, so as to reduce RVSP and reduce mortality of the rats. These findings might provide evidence for the application of notopterol in the therapeutic treatment of PAH.

## Data Availability Statement

The original contributions presented in the study are included in the article/Supplementary Material, further inquiries can be directed to the corresponding author/s.

## Ethics Statement

The animal study was reviewed and approved by Institutional Animal Use and Care Committee of Sun Yat-sen University.

## Author Contributions

LH conceived the study and was responsible for the data acquisition, analysis, and interpretation and wrote the first draft of the paper. HL and SW carried out the experiments and contributed to the data acquisition. SH, QL, LL, SG, GF, and PZ helped with the data analysis, manuscript composition, and proofreading. GC contributed substantially to the conception and design of the study and helped with the data analysis and interpretation. ZW acquired funding, supervised the study and edited the manuscript. All authors read, discussed, commented on and approved the final version of the manuscript. All authors contributed to the study conception and design.

## Funding

This study was supported by the Natural Science Funds of Guangdong Province [Grant No. 2019A1515010218], the National Natural Science Foundation of China [Grant Numbers. 81770319, 82070297], and the National Key R&D Program of China [Grant No. 2017YFC1105000].

## Conflict of Interest

PZ was employed by GuangZhou Janus Biotechnology Co. Ltd. The remaining authors declare that the research was conducted in the absence of any commercial or financial relationships that could be construed as a potential conflict of interest.

## Publisher's Note

All claims expressed in this article are solely those of the authors and do not necessarily represent those of their affiliated organizations, or those of the publisher, the editors and the reviewers. Any product that may be evaluated in this article, or claim that may be made by its manufacturer, is not guaranteed or endorsed by the publisher.

## Abbreviations

PAH: pulmonary arterial hypertension
HPASMCs: human pulmonary arterial smooth muscle cells
RVSP: right ventricular systolic pressure
MCT: monocrotaline
RV: right ventricular.
