# Supplementary material for: Notopterol Attenuates Monocrotaline-Induced Pulmonary Arterial Hypertension in Rat
Source: Front Cardiovasc Med. 2022 Jun 3;9:859422. doi: 10.3389/fcvm.2022.859422 (PMC9203832; doi:10.3389/fcvm.2022.859422)
Supplement: Supplementary file 1 [file Data_Sheet_1.PDF]

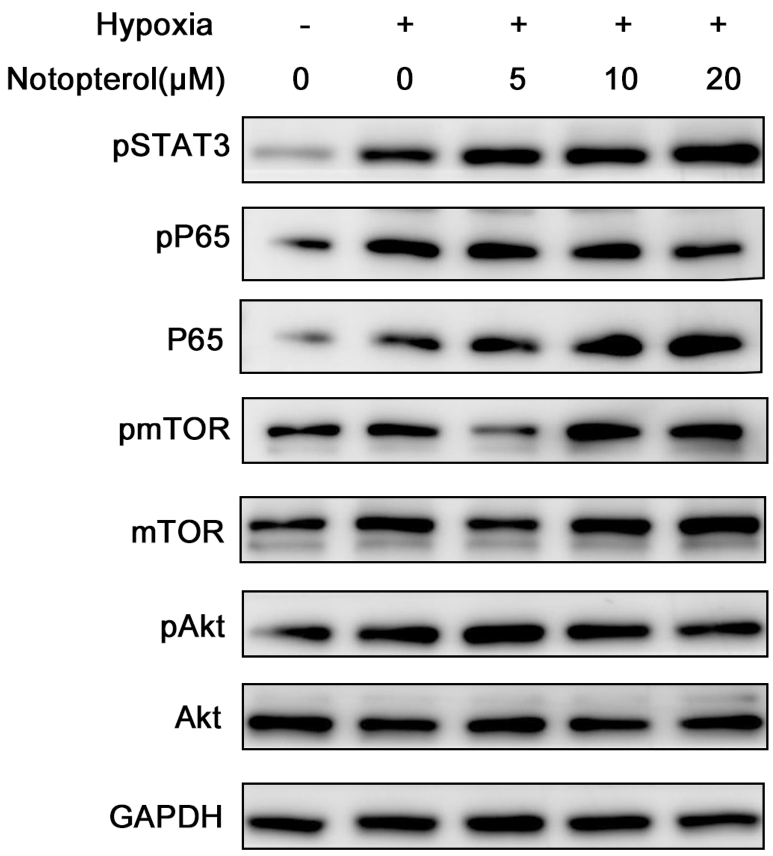

Fig.S1 The effect of notopterol for the expression of Akt/mTOR, p-STAT3 and NF-κB of human pulmonary artery smooth muscle cells in response to hypoxia in vitro
